# A Comparative Study of Iron Uptake Rates and Mechanisms amongst Marine and Fresh Water Cyanobacteria: Prevalence of Reductive Iron Uptake

**DOI:** 10.3390/life5010841

**Published:** 2015-03-11

**Authors:** Hagar Lis, Chana Kranzler, Nir Keren, Yeala Shaked

**Affiliations:** 1 Interuniversity Institute for Marine Sciences in Eilat, Israel; E-Mails: chana.kranzler@mail.huji.ac.il (C.K.); yshaked@vms.huji.ac.il (Y.S.); 2 The Freddy and Nadine Herrmann Institute of Earth Sciences, Edmond J. Safra Campus, Givat Ram, The Hebrew University of Jerusalem, Jerusalem 9190401, Israel; 3 Department of Plant and Environmental Sciences, Institute of Life Science, The Hebrew University of Jerusalem, Jerusalem 91904, Israel; E-Mail: nir.ke@mail.huji.ac.il

**Keywords:** cyanobacteria, iron, siderophore, reduction, phytoplankton, iron uptake

## Abstract

In this contribution, we address the question of iron bioavailability to cyanobacteria by measuring Fe uptake rates and probing for a reductive uptake pathway in diverse cyanobacterial species. We examined three Fe-substrates: dissolved inorganic iron (Fe') and the Fe-siderophores Ferrioxamine B (FOB) and FeAerobactin (FeAB). In order to compare across substrates and strains, we extracted uptake rate constants (k_in_ = uptake rate/[Fe-substrate]). Fe' was the most bioavailable Fe form to cyanobacteria, with k_in_ values higher than those of other substrates. When accounting for surface area (SA), all strains acquired Fe' at similar rates, as their k_in_/SA were similar. We also observed homogeneity in the uptake of FOB among strains, but with 10,000 times lower k_in_/SA values than Fe'. Uniformity in k_in_/SA suggests similarity in the mechanism of uptake and indeed, all strains were found to employ a reductive step in the uptake of Fe' and FOB. In contrast, different uptake pathways were found for FeAB along with variations in k_in_/SA. Our data supports the existence of a common reductive Fe uptake pathway amongst cyanobacteria, functioning alone or in addition to siderophore-mediated uptake. Cyanobacteria combining both uptake strategies benefit from increased flexibility in accessing different Fe-substrates.

## 1. Introduction

Cyanobacteria are a diverse and widespread group of prokaryotes found in a range of marine, fresh, and brackish water environments. Apart from contributing significantly to global primary production [1,2] and nitrogen fixation [3], cyanobacteria influence chemical cycling, ecological structure, and water quality on a regional scale. Due to the high iron content of their photosynthetic apparatus and the role of ferric enzymes in nitrogen fixation, cyanobacteria have particularly high Fe demands relative to heterotrophic bacteria and eukaryotic phytoplankton [4,5,6,7]. Indeed, iron often limits cyanobacterial carbon e.g., [8,9] and nitrogen fixation rates [5] and was shown to influence species composition and cyanobacterial abundance e.g., [10].

Iron is characterized by its exceedingly low solubility in oxic, circum-neutral pH waters, and, as such, Fe rapidly precipitates out of solution as ferric oxyhydroxide species [11]. The colloidal and particulate iron pools (operationally defined as the size fraction >0.02 μm) are not considered directly accessible to phytoplankton, although ongoing studies show that biological and photochemical transformations of these fractions may be an important source of available iron [12] and references therein. The more accessible soluble iron pool (<0.02 μm) is typically found at sub-nanomolar levels [13] and can be roughly divided into two fractions: free inorganic iron (Fe') and organically complexed Fe. While Fe' has proven to be a highly bioavailable Fe substrate to eukaryotic phytoplankton and some cyanobacteria [14,15,16,17], while [13], it found at pM levels, accounting for less than 1% of dissolved iron in surface waters. The overwhelming majority (>99%) of dissolved Fe is bound to organic ligands [18,19,20,21]. However, the chemistry and structures of complex organic ligands are obscure, and thus the bioavailability of this heterogeneous fraction remains poorly defined.

The bioavailability of any particular Fe-substrate depends not only on its chemistry but also on the Fe-uptake mechanisms available to an organism. Different cyanobacterial species may possess different iron uptake strategies. Therefore, iron bioavailability is not only a question of “what?” but also of “to whom?” and “how?” As prokaryotes, cyanobacteria are often associated with the siderophore mediated iron uptake pathways implemented by many heterotrophic bacteria. Siderophores are low molecular weight compounds secreted by iron-limited microorganisms with the purpose of scavenging iron from the environment. These compounds have very high Fe(III) affinities. Once bound to iron, the ferric-siderophore complexes are transported back into the cell via siderophore specific transporters. Decomplexation of the ferric siderophores usually occurs in the cytoplasm [22]. In recent years, the paradigm of siderophore mediated uptake amongst cyanobacteria has been re-evaluated in the face of experimental and genetic studies showing that (a) not all cyanobacteria possess the components of a siderophore based uptake system and (b) alternative Fe-uptake pathways exist in cyanobacteria. Genetic studies demonstrated that siderophore biosynthesis and transporter genes are absent from open ocean cyanobacteria and several fresh water strains e.g., [23,24,25]. Moreover, even in species that are known to produce siderophores, experimental work points to the operation of a *siderophore independent* Fe-uptake pathway [26,27,28,29,30]. Salmon *et al.* [31] reported on the importance of superoxide mediated iron reduction in the cyanobacterium, *Lyngbya majuscula.* Lis and Shaked [32] later proposed that reductive iron uptake directly mediated by the cell may be important in natural populations as well as in open ocean *Synechococcus* species. This reductive strategy is well studied in eukaryotic phytoplankton and involves the reduction of free or complexed ferric iron into its ferrous form prior to its transport across the plasma membrane (either in a re-oxidised ferric or ferrous form) [33,34]. Kranzler and coworkers [16] showed that reduction of Fe(III) plays a central role in the uptake of both Fe' and organically bound iron by the non siderophore producing, fresh water cyanobacterium, *Synechocystis* PCC6803. Recent work on Fe-uptake by mutants of *Synechocystis* PCC6803 yielded a working model for iron reduction and uptake in this organism [35].

In this contribution we aim to quantify the bioavailability of different Fe-substrates to a wide range of cyanobacterial species, and probe their mechanisms of iron uptake. Specifically, we examine the prevalence of the reductive uptake pathway among different cyanobacteria and across different Fe substrates. We combine a qualitative approach addressing whether a specific iron substrate is accessible (yes/no) and how it is taken up (via reduction/siderophore transporter) with a quantitative evaluation of bioavailability by means of cellular Fe uptake rate constants and normalization to surface area.

## 2. Experimental Section

For the purposes of this study, eight strains of cyanobacteria were grown under iron limiting and non-limiting conditions. The growth and iron limitation of each strain were monitored and exponential phase cells were harvested for short-term radioactive iron uptake experiments. Uptake experiments were performed with five different Fe-substrates. We have included the essential methodology in the following section while the finer details can be found in the supplemental.

### 2.1. Trace Metal Clean Techniques

Strict trace metal clean techniques were applied in all culturing and experimental manipulations. Solutions were prepared with double-distilled water (Milli-Q, Millipore, 18.2 mV) and analytical grade chemicals. All work was done under positive pressure HEPA filters. Iron limitation of marine cyanobacteria necessitates very low Fe levels (see Table 1). We thus employed polycarbonate vessels and microwave sterilization in the preparation of growth media and in growth experiments [36]. For fresh water strains, which are easier to iron limit, glassware and autoclave sterilization sufficed. Polycarbonate vessels and glassware were processed as in Shaked *et al.* [34] and Kranzler *et al.* [16].

**Table 1:** The cyanobacterial strains, their media and growth conditions.

Organism ^#^ (abbreviation used in figures)	Brief Description	Siderophore Production	Siderophore Transporters	Diameter * (μm)	Growth Temp (°C)	Growth medium ^§^ [Fe]	Fe-Stress Indicators
***Synechococcus***** WH8102** (WH8102)	Open ocean, unicellular, spherical	No	No	1.2	25	AMP1 0nM (lim) 300 nM (non-lim)	Changes in intracellular photosynthetic pigment ratios (phycocyanin, phycoerythrin and chlorophyll *a*)
***Synechococcus***** WH7803** (WH7803)	Open ocean, unicellular, grown under dim light	No	No	1.2	25	AMP1 0 nM (lim) 300 nM (non-lim)	Changes in intracellular photosynthetic pigment ratios (phycocyanin, phycoerythrin and chlorophyll *a*)
***Synechococcus***** CCMP1183** (CCMP1183)	Open ocean, unicellular	Unknown	Unknown	1.6	25	f/2 0 nM (lim) 300 nM (non-lim)	Decreases in intracellular photosynthetic pigments (chlorophyll *a*)
***Prochlorococcus marinus***** MED4** (MED4)	Open ocean, unicellular	No	No	0.7	25	AMP1 0 nM (lim) 300 nM (non-lim)	Decreases in intracellular photosynthetic pigments (chlorophyll *a*)
***Trichodesmium erythraeum*** (IMS101)	Open ocean, Filamentousdiazotrophic	No	No	Surface area ~157 μm^2 $^	25	YBCII 0 nM (lim) 1 μM (non-lim)	Decreased trichome length
***Synechococcus PCC7002*** ***(PCC7002)***	Brackish water (euryhaline) Coastal	Yes	Yes	1.6	30	A+ 0nM (lim) 1 μM (non-lim)	Decreased growth rate and decreases in intracellular photosynthetic pigments (chlorophyll *a*) and a blue shift in the absorption spectrum
***Anabaena UTEX 2576*** ***(UTEX 2576)***	Fresh water, Filamentous, diazotrophic	Yes	Yes	Surface area ~60 μm2 ^$^	30	YBG11 0.1 μM (lim) 10 μM (non-lim)	Decreases in intracellular photosynthetic pigments (chlorophyll *a*) and a blue shift in the absorption spectrum
***Synechocystis PCC6803*** ***(PCC6803)***	Fresh water, unicellular	No	Putative aerobactin transporter	1	30	YBG11 0.1 μM (lim) 10 μM (non-lim)	Decreases in intracellular photosynthetic pigments (chlorophyll *a*) and a blue shift in the absorption spectrum

Notes: * Diameter when Fe limited was determined microscopically with the exception of *Prochlorococcus* MED4, in which diameter was taken from [37]. For the purposes of calculating cell surface area, all cell geometries were assumed spherical unless otherwise specified. $ Surface area calculated as open cylinder. # Strains were axenic aside from *Trichodesmium* IMS101; § Further details regarding growth media composition can be found the supplemental (Section 11).

### 2.2. Culture Growth and Fe Limitation

The eight cyanobacterial strains in this study vary in their morphology, physiology and environmental origin. Therefore, growth media, iron limiting methodologies, growth curve measurements and detection of iron limitation differed between the strains. Table 1 lists and briefly describes the species and experimental techniques used in growth and iron limitation analysis. All cultures were grown under continuous light to avoid influence of diurnal cycles on short term Fe uptake rate measurements. Growth was monitored using optical density (750 nm; Cary 300Bio UV-VIS spectrophotometer, Santa Clara, CA, USA), with the exception of *Prochlorococcus* in which *in vivo* chlorophyll fluorescence was used as a growth proxy. For all strains, a starting culture was grown under high iron in order to establish high biomass. Cells were then concentrated either by centrifugation (2057 rcf, 10 min) or by gravity filtration onto an EDTA washed 5 μm pore size polycarbonate filter (*Trichodesmium* only). Concentrated cells were washed three times (either on the filter or in a test tube) with iron free medium and then suspended into either iron limiting or iron sufficient mediums. Growth and iron limitation were monitored in both treatments. Indicators of iron stress were species specific and included the measurement of growth rates, *in vivo* adsorption spectra as proxies of photosynthetic pigment content (Cary 300Bio UV-VIS spectrophotometer), the presence of a blue shift in the chlorophyll first excited state maximal absorption wavelength ($\lambda_{max}$) of iron starved cyanobacteria [38,39] and references therein, changes in cell size, and changes in the low temperature chlorophyll fluorescence spectra [39]. Details regarding each indicator of Fe stress and its suitability to the different cyanobacterial strains can be found in the supplemental (Section 2).

### 2.3. Measuring Fe Uptake Rates

We assessed the kinetics and mechanisms of cyanobacterial iron uptake by means of short-term (4–8 h) ^55^Fe uptake experiments. Experiments were performed with ^55^Fe (^55^FeCl_3_, Perkin Elmer, Botston, MA, USA) precomplexed to a chelator. All experimental uptake media comprised of growth media containing no trace metals, nutrients, or vitamins (*i.e.*, salts only). With the exception of YBG11, which contains HEPES, these media contain no organic buffers but rather 2 mM of freshly made trace metal clean NaHCO_3_. The pH in all experimental media ranged between 7.8 and 8.1. Table 2 outlines the species-specific uptake media. For further details regarding media composition see supplemental Section 11.

In Fe' (dissolved inorganic iron) uptake experiments, ^55^Fe was precomplexed to EDTA (Fe: EDTA 1:2) prior to spiking into an EDTA buffered medium. The EDTA buffer maintained a constant and easily calculated Fe' pool throughout the experiment duration (see Section 3 in the supplemental for Fe' calculations). We assume that Fe' is the only iron substrate for uptake in EDTA buffered experiments. While the presence of siderophores in experiments with Fe-limited siderophore-producing strains cannot be entirely ruled out, the buildup of significant siderophore concentrations during the uptake experiment is unlikely given the rigorous washing of cells prior to experiments as well as the short duration of such measurements (see Section 7 in the supplemental for further discussion of this point). It should be noted that EDTA binds Ca^2+^ and Mg^2+^ in the outer membrane of cyanobacteria and, at high enough concentrations, may lead to increased outer membrane permeability [40]. Since different cyanobacteria are sensitive to EDTA at varying degrees, EDTA concentrations in the medium varied with organism (see Table 2). Four siderophores were selected for this study—desferrioxamine B (DFB) (Desferral, Sigma), desferrioxamine E (DFE), aerobactin, and schizokinen (all three from EMC microcollections, Tübingen, Germany). The first two (DFB and DFE) are hydroxamate siderophores which are not known to be produced by marine cyanobacteria and are thus suitable for assessing non-siderophore mediated uptake pathways. Schizokinen is an endogenous siderophore produced by *Anabaena* UTEX2576 and aerobactin is a structural analogue of schizokinen. In FeL uptake experiments (L = DFB, DFE, aerobactin or schizokinen), ^55^Fe was precomplexed to an excess of free ligand prior to spiking into an EDTA-free medium. The presence of excess ligand ensured negligible Fe' concentrations in FeL experiments. An iron to ligand ratio of 1:1.1 was used when complexing Fe to DFB and DFE, while a ratio of 1:3 was used for aerobactin and schizokinen. All precomplexed iron-ligand solutions were adjusted to pH 5–7 using trace metal clean NaOH and then allowed to equilibrate overnight prior to spiking of the experimental medium. After spiking, experimental Fe-uptake media were allowed to equilibrate overnight.

**Table 2:** Short term ^55^Fe uptake experiments—organisms, substrates, and experimental media. Fe' uptake was performed with Fe-limited and non-limited cells. FeL uptake was performed only with Fe-limited cultures. Abbreviations: Fe'—dissolved inorganic iron; FOB—ferrioxamine B; FOE—ferrioxamine E; FeAB—FeAerobactin.

Organism (abbreviations used in text and figures)	Uptake Medium	* EDTA Concentration (μM)	Substrates Tested
***Synechococcus***** WH8102** (WH8102)	AMP1 salts (Turk’s island salt mix) + 2 mM NaHCO_3_	20	Fe', FOB, FeAB, FOE
***Synechococcus***** WH7803** (WH7803)	AMP1 salts (Turk’s island salt mix) + 2 mM NaHCO_3_	20	Fe', FOB
***Synechococcus***** CCMP1183** (CCMP1183)	Synthetic Ocean water (SOW)	20	Fe', FOB, FeAB
***Prochlorococcus marinus***** MED4** (MED4)	AMP1 salts (Turk’s island salt mix) + 2 mM NaHCO_3_	20	Fe', FOB, FeAB
***Trichodesmium erythraeum*** (IMS101)	Synthetic Ocean water (SOW)	20	Fe'
***Synechococcus***** PCC7002** (PCC7002)	A+ salts + 2 mM NaHCO_3_	80	Fe', FOB, FeAB
***Anabaena***** UTEX2576** (UTEX2576)	YBG11	16	Fe', FOB, FeAB, FeSchizokinen
***Synechocystis PCC6803*** (PCC6803)	YBG11	16	FeAB

* Only present in Fe' uptake experiments.

Addition of phytoplankton cells to the uptake medium marked the start of an uptake experiment. Fe' uptake, which is characterized by relatively high uptake rates and thus high signals, was measured in both iron limited and non-limited cultures while FeL uptake, which is characterized by significantly lower uptake rates and signals, was measured only in iron limited cells. Experiments were conducted at growth temperature and in the dark since some of the Fe substrates are photolabile. We found that gentle shaking of the experimental media over the course of the uptake experiment is essential for homogenous results with the filamentous strains *Anabaena* and *Trichodesmium*. At various times during the 4–8 h uptake experiments, weighted volumes of the experiment medium were filtered onto polycarbonate filters or nitrocellulose filters (for strongly coloured cyanobacteria such as *Anabaena*, *Synechococcus* CMMP1183, *Synechocystis* PCC6803, and *Synechococcus* PCC7002, in which colour quenching can interfere with the measurement). Duplicate filtrations were conducted for all time points. Filters were then rinsed with a saline solution, washed with Ti-citrate-EDTA reagent [41] for 2–5 min and then again rinsed with Fe free uptake medium. Nitrocellulose filters were processed as described in Kranzler *et al.* [16] to prevent chlorophyll quenching of signals. Otherwise, filters were placed in Quicksafe A scintillation liquid (Zinsser Analytic, Frankfurt, Germany) and retained for measurement of radioactivity in a Beckman scintillation counter. Intracellular iron was calculated from the average specific activity (activity of the medium divided by the total iron added). Iron uptake rates were calculated from linear regression analysis of intracellular Fe accumulation over time. Uptake rates were normalized to cell counts (if available) and/or extracted chlorophyll-a concentration.

### 2.4. Mechanism of Fe Uptake: the Ferrozine Assay

In the current study, we specifically probed for the reductive iron uptake pathway using the ferrozine assay as described for eukaryotic phytoplankton by Shaked *et al.* [42] and for cyanobacteria by Kranzler *et al.* [16]. This method involves the addition of 200 μM ferrozine to the experimental medium at the start of the experiment. Ferrozine inhibition of uptake is observed only when a ferrous iron intermediate is formed prior to transport across the plasma membrane and occurs in reductive iron uptake but not in uptake via Fe-siderophore transporters. Formation of the Fe(II)Fz_3_ complex is pH sensitive [43] and we thus kept the medium pH between 7.8 and 8.1 (at pH values slightly above pH 8.2 no ferrozine effect was observed, see Section 4 in the supplemental). The presence of organic buffers (e.g., HEPES, TRIS) interfered with the ferrozine effect in some of the media (see Section 4 in the supplemental). Therefore, organic buffers were avoided and replaced with 2 mM NaHCO_3_.

### 2.5. Calculation of Uptake Rate Constants—k_in_

We calculated Fe uptake rate constants in order to compare the uptake of different cyanobacterial species and different Fe substrates. The uptake rate constant (*k_in_*, in units of L cell^−1^·h^−1^) is calculated by dividing cellular iron uptake rate (*ρ*, in units mol Fe·cell^−1^·h^−1^) by the concentration of iron substrate in the medium (*S*, in units mol Fe·L^−1^). See Equation (1):

(1) $$k_{in}=\frac{\rho }{\left[S\right]}$$

While this method enables cross-species comparison, it is a linear approximation and thus applicable for low substrate concentrations (for additional discussion see Lis *et al.* [17]). When iron is completely complexed by an organic chelator (FeL), the substrate concentration, [S], is equal to the total concentration of iron in the medium. However, when working with FeEDTA, it should be noted the FeEDTA complex is not bioavailable [16] and that substrate [*S*] is in fact equal to the concentration of Fe' in the medium. Fe' concentrations are influenced by medium chemistry, especially by the presence of Mg^2+^ and Ca^2+^ ions. Therefore, additional short-term uptake experiments were performed with *Synechococcus* species in different experimental media to verify Fe' calculations in a number of differently composed media (see supplemental Section 3 for results and calculations). Fe' concentrations were calculated using Visual Minteq software [44].

## 3. Results

### 3.1. Fe' Uptake

**Figure 1:**
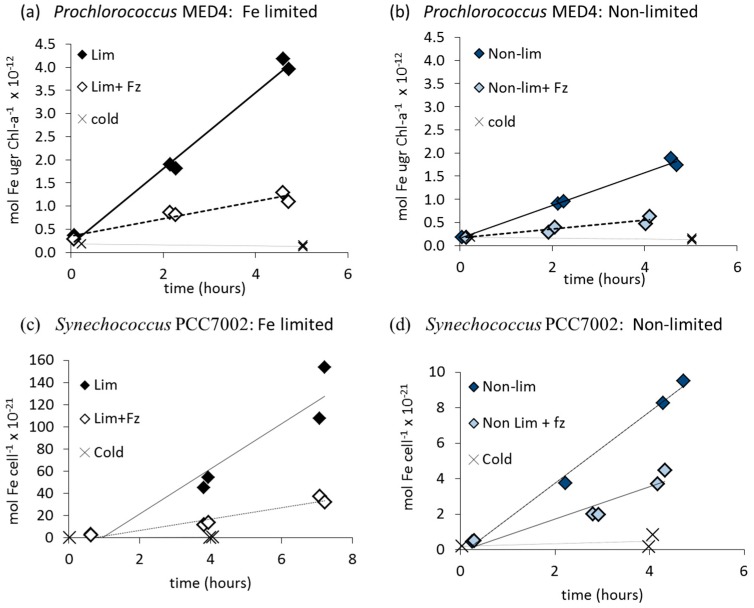
Accumulation of intracellular dissolved inorganic iron (^55^Fe') over time in short term iron uptake experiments in an EDTA buffered medium for Fe limited (Lim) and non Fe-limited (Non lim) cells in the absence and presence of 200 μM ferrozine (Fz). Fe' uptake by cells at 4 °C (cold controls) is also shown. (**a**,**b**) Open ocean, non siderophore producing *Prochlorococcus* MED4 (20 μM EDTA, 60 nM FeEDTA for limited cells, 20 μM EDTA, 80 nM FeEDTA for non-limited cells); (**c**,**d**) Brackish water, diazotrophic, siderophore producer *Synechococcus* PCC7002 (80 μM EDTA, 90 nM FeEDTA for limited cells, 90 nM FeEDTA for non-limited cells). Note that uptake rates are normalized to either Chl-a or cell number, depending on the cyanobacterial species.

We studied the uptake rates and mechanisms of dissolved inorganic iron (Fe') by seven strains of cyanobacteria. Under both iron limiting and non-limiting conditions, all strains exhibited linear Fe' uptake with correlation coefficients typically >0.8. Figure 1a,b (*Prochlorococcus* MED4) and 1c,d (*Synechococcus* PCC7002) show examples of short-term intracellular ^55^Fe accumulation from an EDTA-buffered medium. Fe' uptake rates differed markedly between cells which were acclimated to Fe-limitation (Figure 1a,c) and those grown under Fe-sufficient conditions (Figure 1b,d), with uptake rate constants of all strains increasing by an order of magnitude under iron limitation, with the exception of *Anabaena* UTEX2576 (see supplemental Table S3).

Regardless of whether they were iron limited or not, the addition of the ferrous iron Fe(II) chelator, ferrozine (Fz), inhibited Fe' uptake in all strains (Figure 1; Table 3). These results show that a ferrous iron intermediate is formed in the process of Fe' uptake and thus imply that reduction plays a central role in the acquisition of Fe' by a range of cyanobacterial species under Fe-limiting and Fe sufficient conditions alike.

**Table 3:** Inhibition of short-term cyanobacterial ^55^Fe uptake by ferrozine (FZ) indicates whether a reductive Fe uptake pathway is at play. Significant inhibition of uptake by FZ is indicated by “Yes,” while lack of inhibition or inconclusive data are indicated by “No” and “n/a,” respectively. Inhibition of uptake is considered significant or not according to the error on linear regression analysis on short-term uptake. The degree of inhibition is indicated by + signs [(+) 20%–50%; (++) 50%–70%; (+++) >70% inhibition, respectively]. A dash sign (-) indicates that ferrozine effect was not determined.

Organism	Fe' (Free Inorganic Iron)		FOB	FeAB
	Not Limited	Fe-Limited	Fe-Limited	Fe-Limited
***Synechococcus* WH8102**	Yes (+)	Yes (+++)	Yes (++)	n/a
***Synechococcus* WH7803**	Yes (+++)	Yes (+++)	Yes (++)	n/a
***Synechococcus* CCMP1183**	Yes (++)	-	-	-
***Synechococcus* PCC7002**	Yes (++)	Yes (+++)	Yes (+)	No
***Prochlorococcus* MED4**	Yes (++)	Yes (+++)	Yes (+)	n/a
***Synechocystis* PCC6803**	Yes (+++)^a^	Yes (+++)^a^	Yes (++)^a^	Yes (++)
***Trichodesmium* IMS101**	-	Yes (+)	-	-
***Anabaena* UTEX2576**	Yes (+++)	Yes (+++)	No*	No

Note: a- data taken from Kranzler *et al.* [16]. * Data inconclusive.

**Figure 2:**
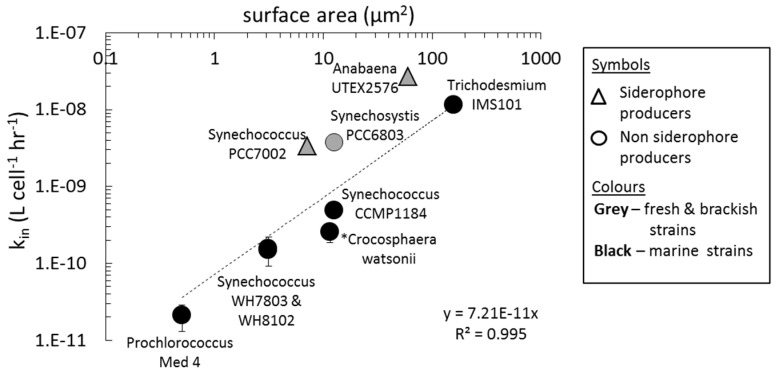
Dissolved inorganic iron (Fe') uptake rate constants (k_in_ = uptake rate/ [Fe']) of Fe-limited cyanobacteria as a function of cell surface area (μm^2^) on a log-log plot. Each data point represents averaged rate constants from a single study for a single organism. Due to ionic strength differences in the media, only marine species are included in the linear regression analysis. * *Crocosphera* *watsonii* WH8501 data was taken from Jacq *et al.* [46]; all other data points were taken from studies conducted in our laboratory. Uptake rates for *Anabaena* and *Prochlorococcus* were normalized to per cell using conversion factors of 158 and 1.4 fg·Chl-a cell^−1^ respectively.

In order to allow comparison between experiments conducted at different iron concentrations, we normalized the uptake rate to [Fe'] (see Equation (1)). The resultant Fe' uptake rate constants (*k_in_*) of Fe-limited cyanobacteria extend over two orders of magnitude. Very large species such as *Trichodesmium* and *Anabaena* exhibit rate constants of ~10^−8^ L·cell^−1^·h^−1^, while the much smaller open ocean *Prochlorococcus* and *Synechococcus* species exhibit constants of ~10^−10^ L·cell^−1^·h^−1^. To examine whether variation in uptake rate constants reflect a difference in the uptake abilities of the various strains or simply a difference in their sizes, we plotted k_in_ values for Fe' as a function of cell surface area (Figure 2). Data points for fresh and brackish water strains (grey symbols) fall above those for marine strains (black symbols). The difference between the two groups may be put down to the higher chemical activity of Fe' in the lower ionic strength media. We estimate that the presence of major ions such as sulphate and chloride at high concentrations in synthetic sea water media may affect the availability of Fe' at the cell surface, resulting in slightly decreased k_in_ values as opposed to the fresh water media. Normalization to the activity coefficients according to Millero and Pierrot [45] greatly reduces scatter between seawater and fresh water data points and places them all along the same straight line (Figure S5). These differences notwithstanding, Figure 2 shows a linear correlation between the cell surface areas and Fe' uptake rate constants of the marine cyanobacterial strains. Forcing the trend line through the origin changes neither the slope nor the correlation co-efficient significantly (y = 7.210 × 10^−11^, r^2^ = 0.99), indicating direct proportionality between cell surface area and the Fe' uptake rate constants of cyanobacteria.

### 3.2. Ferric Siderophore Uptake

#### 3.2.1. Ferrioxamine B (FOB)

**Figure 3:**
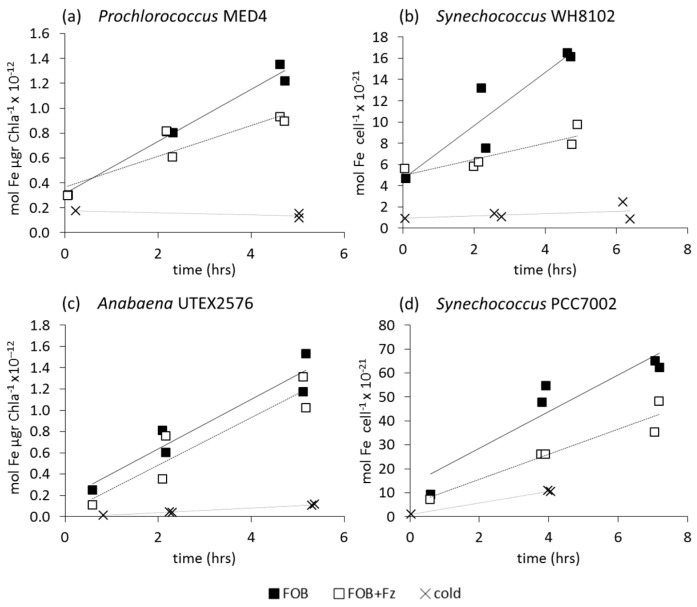
Accumulation of intracellular iron in short term ferrioxamine B (FOB) uptake experiments in the absence (black squares) and presence (white squares) of 200 μM ferrozine (Fz) by Fe-limited cells of two non siderophore producing (a,b) and two siderophore producing cyanobacterial strains (c,d). Fe' uptake by cells at 4 °C (cold controls) is also shown (x symbols). (**a**) *Prochlorococcus* MED4 (63nM FOB); (**b**) *Synechococcus* WH8102 (64 nM FOB); (**c**) *Anabaena* UTEX2576 (60 nM FOB); (**d**) *Synechococcus* PCC7002 (88 nM FOB). Note that uptake rates are normalized to either Chl-a or cell numbers, depending on the cyanobacterial species.

We measured ^55^FOB uptake rates only in iron-limited cells. Like Fe', intracellular ^55^Fe accumulation from the FOB complex by iron-limited cells over time is linear in all strains with correlation coefficients, typically >0.8 (Figure 3). The addition of 200 μM Fz inhibited ^55^FOB uptake in all cyanobacteria (Figure 3, Table 3), with the exception of *Anabaena*, in which a slight inhibitory effect was only sometimes observed (Figure 3c). Therefore, reduction plays a central role in the uptake of iron from the FOB complex by a wide range of cyanobacteria.

**Figure 4:**
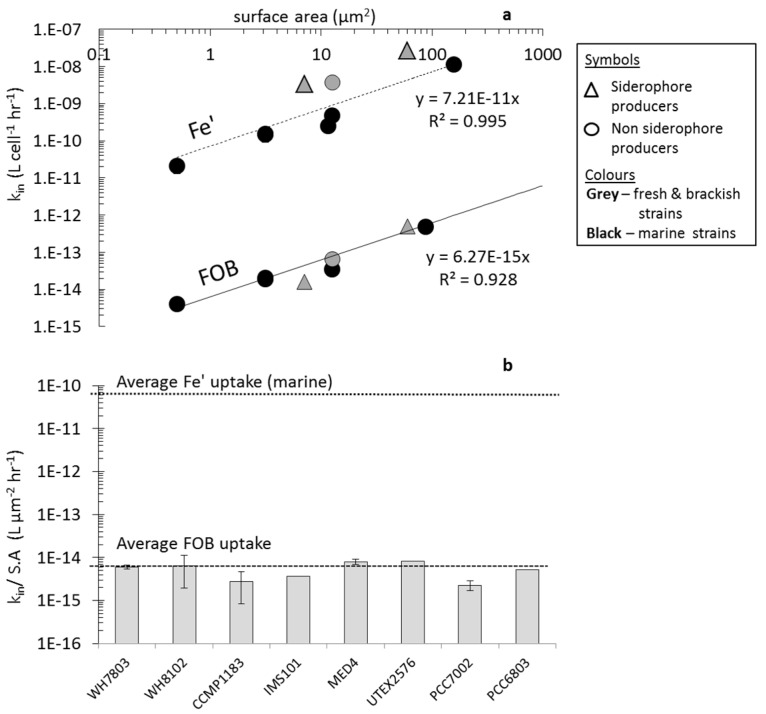
A comparison of FOB and Fe' uptake by iron limited cyanobacteria. (**a**) Uptake rate constant (k_in_ = uptake rate/ [Fe-substrate]) as a function of cell surface area on a log-log plot. Linear regression analysis on Fe' includes only marine strains, while that for FOB includes all strains; (**b**) Species-specific FOB uptake data. In order to compare between species, we normalized the uptake rate constant to cell surface area (*i.e.*, k_in_/S.A.). The dashed lines indicate the average k_in_/SA of Fe' and FOB uptake for all species *i.e.*, the slope of the Fe' and FOB trend lines in Figure A. Uptake rates were normalized to per cell for *Anabaena* and *Prochlorococcus* using conversion factors of 158 and 1.4 fg·Chl-a·cell^−1^, respectively.

Similarly to Fe' uptake, the uptake constants (k_in_) of FOB (where k_in_ = uptake rate/[FOB]), are linearly correlated to cell surface area for a range of species (Figure 4a). Figure 4b summarizes the surface area normalized uptake rate constants (in units of L μm^−2^·h^−1^) of a range of cyanobacterial species from the current study as well as previously published works and emphasizes the appreciable similarity in FOB k_in_/S.A. values across different cyanobacterial strains. For both Fe' and FOB all experimental cyanobacterial strains fall along a linear regression line (Figure 4a). Therefore, we can extract the slope of the two trend lines defining Fe' and FOB uptake in order to compare the bioavailability of these two substrates on a cell surface area basis. The average FOB k_in_/S.A. value as indicated by the slope of the linear trend line (6.3 × 10^−15^ L·μm^−2^·h^−1^) is four orders of magnitude lower than that for Fe' (7.21 × 10^−11^ L·μm^−2^·h^−1^). A summary of all measured ^55^FOB uptake rate constants can be found in the supplemental (Table S3).

#### 3.2.2. Fe-Aerobactin (FeAB)

**Figure 5:**
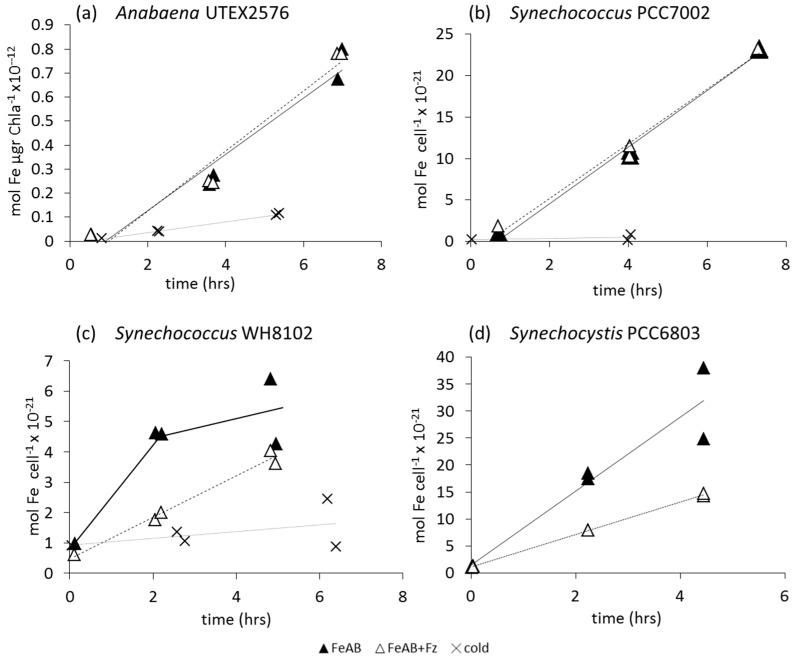
Short-term ^55^FeAerobactin uptake in the absence (black triangles) and presence (white triangles) of 200 μM ferrozine (Fz) by iron limited cells of two siderophore (a,b) and two non-siderophore producing (c,d) cyanobacteria. Fe' uptake by cells at 4 °C (cold controls) is also shown. (**a**) *Anabaena* UTEX2576 (87nM FeAB) (**b**) *Synechococcus* PCC7002 (87 nM FeAB) (**c**) *Synechococcus* WH8102 (68nM FeAB) and (**d**) *Synechocystis* PCC6803 (150 nM FeAB).

In contrast to FOB and Fe', FeAB exhibits variability in both rate and mechanism of uptake by the different cyanobacterial strains. Linear uptake kinetics of this compound can be observed in the two siderophore producers, *Anabaena* UTEX 2576 and *Synechococcus* PCC7002 (Figure 5a,b), as well as in the non siderophore producing, fresh water, *Synechocystis* PCC6803 (Figure 5d). In the open ocean cyanobacterium, *Synechococcus* WH8102, the uptake rate is not linear and slows over time (Figure 5c). Similar trends were seen in several other open ocean strains (Figure S6) and we hypothesize that this data reflects adsorption rather than true uptake of FeAB into the cell (see supplemental Section 8).

Addition of ferrozine to the uptake medium of open ocean strains had no specific or consistent effect (e.g., Figure 5c and S6). Ferrozine did not inhibit ^55^FeAB uptake rates by both *Anabaena* UTEX 2576 and *Synechococcus* PCC7002 (Figure 5a,b, Table 3), suggestive of a non-reductive uptake pathway. In accord, ferrozine also did not inhibit the uptake of the endogenous FeSchizokinen in *Anabaena* (see supplemental Figure S9). Since both *Anabaena* and *Synechococcus* PCC7002 synthesize siderophores, which bear a strong structural similarity to FeAB [47,48], FeAB may be taken up by siderophore specific transporters. In contrast to these findings, ferrozine does inhibit FeAB uptake by the non-siderophore producer, *Synechocystis* PCC6803 (Figure 5d, Table 3).

**Figure 6:**
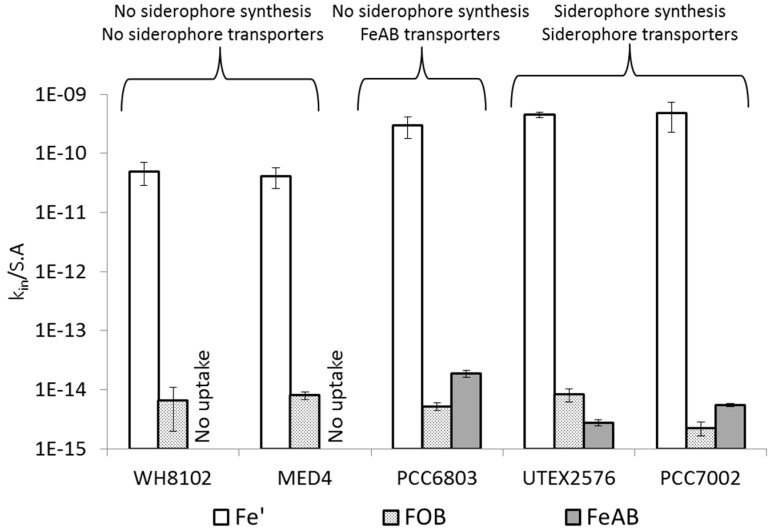
^55^FeAerobactin (FeAB) uptake as compared to Fe' and FOB uptake by five representative strains of iron limited cyanobacteria. In order to compare between species, uptake rate constants (k_in_) were normalized to cell surface area (*i.e.*, k_in_/S.A in units of L·μm^−2^·h^−1^).

Comparison of FeAB uptake across organisms can be made after normalization of the uptake rate constants to cell surface area (k_in_/S.A)—see Figure 6. Relative to Fe' and FOB uptake, three distinct behaviors can be seen in FeAB uptake: (1) No uptake amongst marine strains; (2) more efficient uptake than FOB in *Synechocystis* PCC6803, and (3) FeAB which is similar to or lower than FOB uptake in *Synechococcus* PCC7002 and *Anabaena* UTEX2576.

## 4. Discussion

### 4.1. Reductive Uptake of Fe' and FOB is Shared by All Studied Strains

Results show that ferrozine inhibited the uptake of dissolved inorganic iron (Fe') in all studied cyanobacterial strains (Figure 1, Table 3). Studies on *Synechocystis* PCC6803 show that Fe(II)Fz_3_ formation is of the same order of magnitude as cellular Fe' uptake rates [16], further strengthening the link between reduction and iron uptake. It is interesting to note that ferrozine inhibited the Fe' uptake of iron limited and non-limited cells (Figure 1, Table 3), meaning that the reductive mechanism serves cyanobacteria in both iron deficient and sufficient environments. As opposed to the labile Fe' species, ferric siderophore complexes pose a considerable challenge to the reductive Fe uptake pathway in that the siderophore-bound iron is extremely stable and therefore difficult to extract. Furthermore, ferric siderophore complexes are characterized by negative redox potentials −350 mV to −750 mV/NHE; [49]. Nonetheless, with the exception of *Anabaena*, FOB uptake by all strains was clearly inhibited by ferrozine indicating that like Fe', FOB uptake by cyanobacteria proceeds via a reductive mechanism. We thus propose that reductive uptake is a prevalent iron uptake strategy amongst both cyanobacteria and other phytoplankton [15] and references therein which may serve in the acquisition of a wide range of substrates ranging from free inorganic iron to strongly bound Fe species.

### 4.2. Uptake of Fe' and FOB: Similarities amongst Diverse Cyanobacterial Species

Comparison of Fe' bioavailability to different cyanobacteria reveals a high degree of similarity between nine distinct strains as shown by the robust relationship between the Fe' uptake rate constants and the cell surface areas of all strains in Figure 2. In terms of bioavailability, it is important to note that Fe' is four orders of magnitude more available than FOB (Figure 4a; Table S3). This is probably due to ease of passage through the outer membrane (Fe' is small while FOB is bulky) and due to ease of reduction (Fe' is labile while FOB is stable). Despite its vastly different chemistry, FOB uptake also shows linear proportionality between the uptake rate constant and surface areas of five cyanobacterial strains (Figure 4a). The k_in_—surface area relationship observed here is surprising in view of the profound morphological (small and large, single celled or filamentous), physiological (diazotrophs and non-diazotrophs; siderophore and non-siderophore producers) and environmental (fresh, brackish and marine waters) differences between the organisms. Similarity in iron uptake efficiency per surface area across diverse cyanobacterial species implies that strong selective pressures have led to the development of a common iron uptake mechanism amongst cyanobacteria. The results of ferrozine assays strongly suggest that this common mechanism is reduction.

### 4.3. Uptake of FeAB—a Case Study in Differential Bioavailability

As opposed to Fe' and FOB, FeAB uptake by cyanobacteria shows little uniformity amongst the different strains. FeAB thus presents a case study in which to explore how both iron chemistry and physiological iron uptake systems influence the resultant bioavailability of a given Fe substrate. The FeAB complex is characterized by a net molecular charge of −3 which appears to be a key determinant of it bioavailability due to its electrical repulsion from the negatively charged cyanobacterial outer membrane. Indeed, cyanobacterial strains which lack siderophore transporters (*i.e.*, open ocean *Synechococcus* and *Prochlorococcus* species), have very little or no access to the FeAB complex (Figure 5c, see supplemental Section 8 for further discussion). However, these organisms were able to take up iron from the positively charged FOB (Figure 6) and at a slower rate from the electrically neutral ferrioxamine E complex (FOE) (Figure S10). Strains which produce siderophores similar in structure to FeAB—*Anabaena* and *Synechococcus* PCC7002—may be able to access this compound by means of a dedicated siderophore transporter (Figure 5 and Figure 6). Nonetheless, FeAB uptake rates in *Anabaena* UTEX2576 remained low as compared to the uptake of endogenous siderophores (k_in_ = 1.7 × 10^−13^ L·cell^−1^·h^−1^ for FeAB as opposed to 3.3 × 10^−11^ L·cell^−1^·h^−1^ for FeSchizokinen), presumably due to variation in chemical structure and the negative molecular charge of FeAB. *Synechocystis* PCC6803, the only organism possessing putative FeAB specific transporters in its outer membrane [50,51], also exhibited the highest surface area normalized FeAB uptake rate constant out of all the tested strains (Figure 6). We suggest that in the absence of a transporter in the inner plasma membrane, FeAB is not able to cross from the periplasm directly into the cytoplasm. Instead, iron can be released from the FeAB complex in the periplasm via a reductive mechanism [16].

### 4.4. Siderophore vs. Reductive Iron Uptake Pathways: Advantages and Disadvantages

In dilute aquatic environments, reductive iron uptake has several distinct advantages over the siderophore-mediated Fe acquisition strategy. Unless anchored to the cell membrane by a lipophilic tail, rapid diffusion of secreted siderophores away from the cell reduces the likelihood of recapturing iron loaded siderophore complexes [52,53]. In addition, the metabolic costs of siderophore biosynthesis and transport are high, especially when considering the high degree of specificity characterizing siderophore transport systems. In contrast, since cyanobacteria can reduce extremely stable Fe-complexes such as FOB, the reductive pathway may be harnessed in the uptake of diverse Fe complexes, without necessitating the biosynthesis of a range of complex-specific transporters on the outer membrane. A flexible and economical strategy such as reduction is well suited to the heterogeneity of iron speciation in aquatic environments in which chemical Fe species range from unbound inorganic iron, Fe', to Fe complexed to organic ligands of varying strengths including humic substances, exopolysaccharides (EPS), heme-like compounds, and siderophores [19]. We therefore suggest that the reductive iron uptake strategy is a communal solution to the constraints on iron bioavailability and uptake faced by cyanobacteria in aquatic environments.

Reduction is an essential uptake pathway in open ocean cyanobacterial strains, which possess neither siderophore biosynthesis nor transport genes. In siderophore producers, reduction is complemented by additional Fe uptake pathways, conferring them with the advantage of multiple iron uptake systems able to access a wider range of Fe substrates under both iron deficient and sufficient conditions. A growing body of evidence suggests that non-siderophore-producing cyanobacteria may still retain the capacity to transport siderophores [50,51,54] and even implement reduction in order to release the siderophore bound iron. *Synechocystis* PCC6803 may be an example of such a “mix and match” strategy, transporting Fe-siderophores into the periplasm and then releasing the iron from the complex via reduction [35]. Why then don’t all cyanobacteria have both reductive and siderophore based Fe uptake systems? One possible explanation is genome streamlining—the evolutionary maintenance of a compact genome size aimed at minimizing the metabolic cost of replicating DNA, which has little or no adaptive value. For example, *Synechococcus* and *Prochlorococcus* strains adapted to living in a steady open ocean environment have little need for metabolic flexibility and thus maintain limited core metabolic abilities [55].

### 4.5. Cyanobacterial Uptake Rates and Mechanisms: Implications in Natural Environments

Our results demonstrate that inorganic iron, Fe', can support significantly higher growth rates and intracellular iron quotas in cyanobacteria than equivalent concentrations of FOB or FOB-like compounds. In fact, Fe' (k_in_ = 2.7 × 10^−8^·L·cell^−1^·h^−1^) is one thousand fold more bioavailable to *Anabaena* than its endogenous siderophore Fe-schizokinen (k_in_ = 1.2 × 10^−11^ L·cell^−1^·h^−1^). This can be put down to the chemical lability and small size of Fe' relative to the strongly chelated and bulky Fe siderophore complexes. Siderophore independent access to Fe' would be advantageous in several environmentally relevant situations. In Fe-rich environments, siderophore biosynthesis is reduced [28] and the photochemical cycling of iron produces a transient but significant Fe' pool [56]. Symbiotic interactions between autotrophic and heterotrophic bacteria may lead to a local increase in Fe' levels due to release of photolabile siderophores e.g., [57]. Lastly, in Fe poor environments particulate Fe from aeolian sources may supply some Fe' [12,58].

A linear correlation between the uptake rate constant and surface areas of different cyanobacterial species was found in the uptake of Fe' and FOB. Eukaryotic phytoplankton also show a robust linear k_in_-surface area relationship in the uptake of both these iron substrates [17,59]. This observation suggests that the biological, chemical and physical constraints on iron acquisition in aquatic environments has pushed iron uptake amongst phytoplankton to its optimal efficiency, *i.e.*, the iron uptake rates via the reductive pathway at sub-saturating substrate concentrations cannot increase further [17]. If cyanobacteria acquire Fe substrates at maximal efficiency, then competition between them will be not be dictated by their uptake rate but rather by other factors such as: reducing Fe intake by means of reduction in size, pigment content, or the substitution of iron with other catalytically active metals [60], symbiotic association with heterotrophic bacteria e.g., [57] or the ability to access unconventional iron sources e.g., particulate and colloidal iron [61].

## 5. Conclusions

Our analysis of iron uptake rates by a diverse range of cyanobacteria supports the wide spread implementation of reduction as an iron uptake strategy, regardless of the presence of siderophore mediated uptake pathways. This is reflected in the similarity in k_in_/S.A values in the uptake of Fe' and FOB. Under Fe limitation, reductive Fe uptake appears to operate at its maximal efficiency; hence, a competitive advantage in Fe acquisition can be gained by reducing iron demands and combining multiple uptake pathways.

## Supplementary Files

Supplementary File 1
