# Supplementary material for: A Comparative Study of Iron Uptake Rates and Mechanisms amongst Marine and Fresh Water Cyanobacteria: Prevalence of Reductive Iron Uptake
Source: Life (Basel). 2015 Mar 11;5(1):841–60. doi: 10.3390/life5010841 (PMC4390881; doi:10.3390/life5010841)
Supplement: Supplementary File 1 [file life-05-00841-s001.pdf]

Supplementary material

Table of Contents

Section 1: Trace metal clean techniques 3
Section 2: Iron stress indicators 3
Figure S1: Growth of *Synechococcus* PCC7002 at different [Fe] 4
Figure S2: Changes in pigment content of *Synechococcus* WH8102 under Fe limitation 5
Figure S3: Changes in absorption spectra of *Synechococcus* PCC7002 under Fe limitation 5
Section 3: Fe' calculations 6
Table S1: [Fe'] and k_{in} values in different experimental media 7
Section 4: Influence of organic buffers and pH on the ferrozine assay 7
Figure S4: Ferrozine assay- influence of organic buffers and pH 8
Section 5: Iron chelators: chemical characteristics 9
Table S2: Chemical characteristics of Fe chelators used in this study 9
Section 6: Fe' uptake and ionic strength 10
Figure S5: Normalization of Fe' k_{in} to activity co-efficient 10
Section 7: Presence of siderophores in uptake experiments with siderophore producers 10
Section 8: Fe-aerobactin uptake 12
Figure S6: FeAB uptake by marine cyanobacteria 12
Figure S7&S8: Evidence of repulsion of FeAB from the cell surface of cyanobacteria 13
Section 9: Uptake of Fe-schizokinen and Ferrioxamine E 14
Figure S9: Fe-schizokinen uptake by *Anabaena* UTEX2576 14
Figure S10: Ferrioxamine E uptake by *Synechococcus* WH8102 14
Section 10: Comparison of Fe uptake across substrates and organisms 15
Table S3: Uptake rate constants for all organisms and substrates 15
Section 11: Growth and uptake media composition 16
Table S4: Growth media composition 16
Section 12: Titanium wash 18
References 19

1. Trace Metal Clean Techniques

All polycarbonate vessels used for culture growth and uptake experiments were soaked in soap for 48 h, rinsed in deionised water, followed by a 48 h soak in 10% HCl and a finally rinsed three times with Milli-Q water. After HCl treatment, bottles were filled with Milli-Q water amended with EDTA and stored. All vessels were washed three times with Milli-Q water prior to use. Glass ware was soaked in 10% HCl for at least 48hours, washed thoroughly with DDW and autoclaved prior to use. Plastic ware and salt solutions for marine and brackish strains were microwave sterilised while vitamin, trace metal and nutrient additions were filter sterilized through an acid washed 0.2 μm filter. Glass ware and salt solutions for fresh water strains were autoclaved while vitamin, trace metal and nutrient additions were

filter sterilized through an acid washed 0.2 µm filter. All tips were washed three times with microwave sterilized 10% HCl and then three times with microwave sterilized Milli-Q water prior to use.

2. Iron Stress Indicators

Below we outline the methods employed in this study and their pros and cons. Representative results for each method are shown below.

(1). **Growth rate**—some cyanobacterial groups show a clear decrease in growth rate upon iron limitation. For example, *Synechococcus* PCC7002, a brackish water strain, grows at 0.26 ± 0.04 day^{-1} on 10 µM Fe but at 0.16 ± 0.01 day^{-1} at 0 Fe (Figure S1). However some cyanobacteria, most notably the open ocean strains may not show clear cut decreases in growth rates under iron stress and therefore a more explicit means of detecting Fe limitation should be used in conjunction to measurement of growth rate.

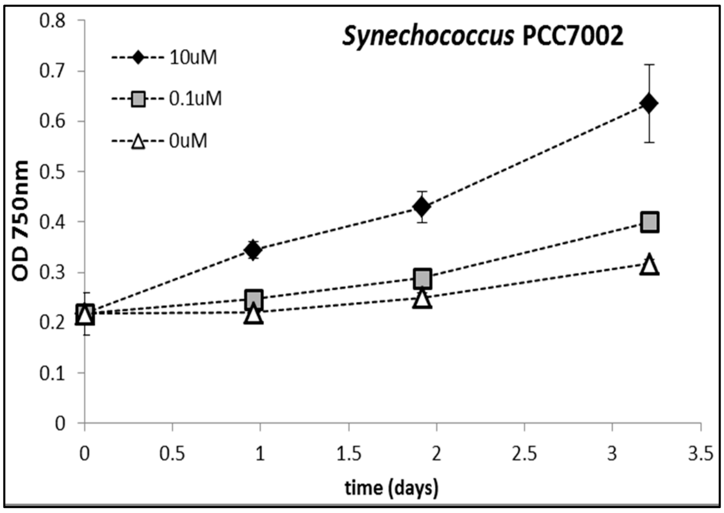

Figure S1. *Synechococcus* PCC7002 grown in 0 (white triangles), 0.1 µM (grey squares) and 10 µM Fe (black diamonds). Growth rates were computed from logarithmic growth phase. The growth medium A^+ contains 80uM EDTA, allowing easy trace metal buffering.

(2). A decrease in photosynthetic pigment content is one of the most noticeable symptoms of iron deprivation in photosynthetic organisms including cyanobacteria (Guikema and Sherman 1983; Öquist 1974). Phycocyanins ($\lambda_{max} = 625\ nm$) are the first to decrease followed by chlorophyll-a ($\lambda_{max} = 678nm$). These changes can readily be followed by the periodic measurement of absorption spectra. See figure S2a for absorption spectra of iron limited and non-limited *Synechococcus* WH8102 cells. Photosynthetic pigment content can be approximated by conducting pigment extractions and cell counts. For example, in *Synechococcus* WH8102 we found 2.73 ± 0.14 fg Chl-a $cell^{-1}$ in iron limited cells (Fe-lim) as opposed to 3.9 ± 0.2 fg·Chl-a $cell^{-1}$ in non-limited (Fe-rep) cultures (Figure S2b).

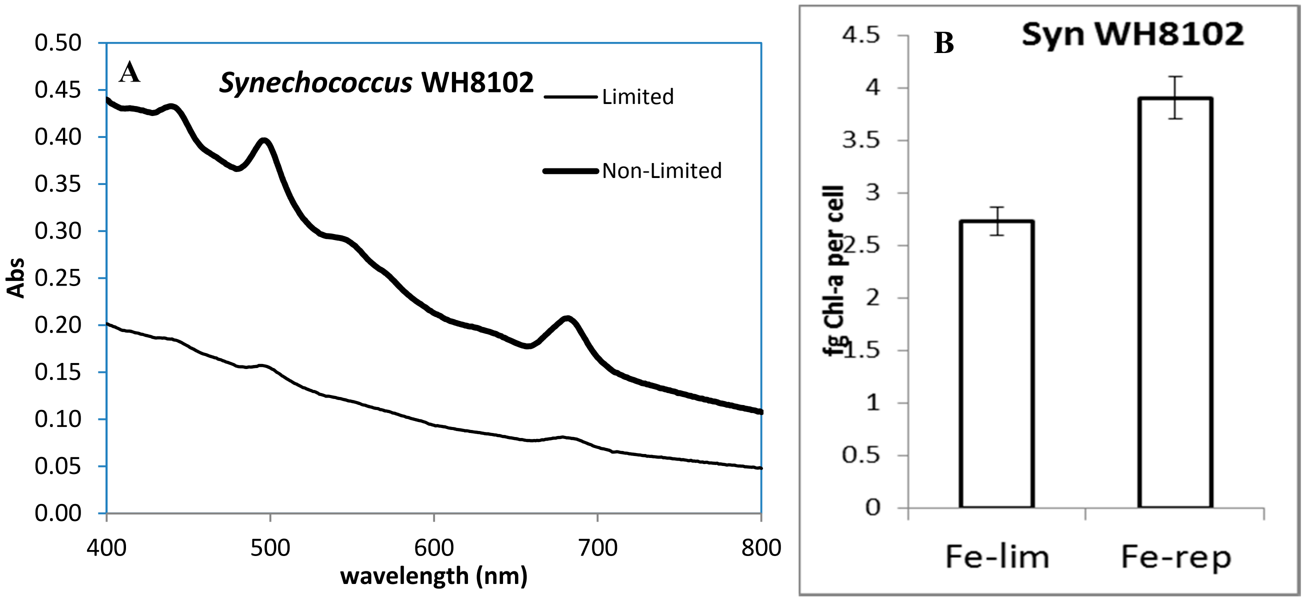

Figure S2. Changes in pigment content due to iron limitation. (**A**) *In vivo* absorption spectra of iron-limited and non-limited *Synechococcus* WH8102 cells after 66 h of growth. Decrease in chlorophyll-a concentrations are evident at ~680 nm and ~430 nm (**B**) Intracellular Chlorophyll-measurements in iron-limited and non-limited *Synechococcus* WH8102 cells as measured from acetone pigment extraction.

(3). A **blue shift** in chlorophyll λ_{max} (maximal absorption wavelength) has previously been characterized in iron starved cyanobacteria (Guikema and Sherman 1983 and references therein). The shift becomes more pronounced as iron limitation intensifies. The shift is particularly evident in the fresh and brackish water cyanobacteria (see Figure S3).

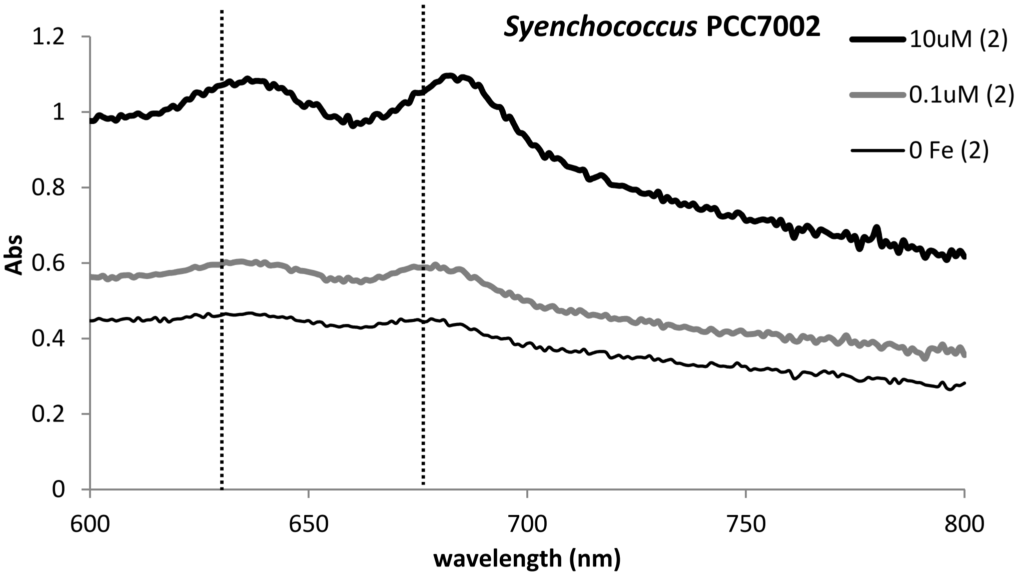

Figure S3. Absorption spectra of Synechococcus PCC7002 cultures after 3.2 days in 10 µM (thick black line), 0.1 µM (grey line) and 0 µM Fe (thin black line). Pigment absorption peaks are indicated by dotted vertical lines in order to better illustrate the blue shift in Fe-limited cultures.

(4). Changes in fluorescence spectra occur in Fe-limited cyanobacteria which can be detected using low temperature (77K) chlorophyll fluorescence spectra measurements which probe for the induction of the iron stress responsive protein, IsiA. This is reflected by an increase in the fluorescence intensity at 682 nm (Singh and Sherman 2007). See Kranzler *et al.* (2011) for an example with *Synechocystis* PCC6803. This technique can supplement growth data and absorption spectra and works best with fresh water cyanobacteria. Caution must be exercised in the application of this method to different cyanobacteria since we found that isiA is also induced in non-limited marine cyanobacterial strains.

(5). A decrease in cell size often characterizes iron limitation amongst photosynthetic microorganisms. Size changes may be difficult to follow in the very small *Synechococcus* and *Prochlorococcus* strains by use of light microscopy alone. Sensitive flow cytometry techniques may be required.

3. Fe' Calculations

The synthetic chelator ethylenediaminetetraacetic acid (EDTA) is commonly used to buffer constant and easily calculable Fe' concentrations (Sunda *et al.* 2005). FeEDTA itself is not bioavailable to eukaryotic phytoplankton(Shaked *et al.* 2005) and does not appear to be bioavailable to cyanobacteria as evidenced from work with *Synechocystis* PCC6803 (Kranzler *et al.* 2011) and the linear relationship between Fe' and uptake rates seen in this work. Thus, in a system containing FeEDTA and sufficient free EDTA, the only bioavailable substrate is Fe'.

In order to calculate the uptake rate constant, k_{in}, it is critical to know Fe' concentration in the experimental medium. This calculation must take into account light intensity (the FeEDTA complex is photolabile) as well as the experimental medium's ionic composition (EDTA binds divalent cations such as Ca^{2+} and Mg^{2+} and therefore their presence in the experimental medium greatly influences final Fe' concentrations) and pH. All experiments in this study were performed in the dark. Visual Minteq softaware (Gustafsson *et al.* 2010) was used to calculate Fe' concentrations in the different experimental mediums. Fe' was calculated as the sum of the following iron species: $Fe(OH)^{2+}$, $Fe(OH)_{3\ (aq)}$ and $Fe(OH)^{4-}$. Calculations for synthetic ocean water were verified using the equations and constants specified by (Sunda and Huntsman 2003). In addition, we performed short term ^{55}Fe' uptake experiments with *Synechococcus* WH8102 and *Synechococcus* PCC7002 in a number of different mediums and compared the resultant k_{in} values—see Table S1 below.

Table S1. Experiments performed with Synechococcus species in different mediums. Each medium has a unique ionic composition and pH and as such, Fe' concentrations as calculated by Visual Minteq differ. In order to verify Fe' calculations, k_{in} values were calculated. Similarity in k_{in} verified Fe' calculations.

Organism	Medium	[EDTA] (μM)	[FeEDTA] (nM)	Fe' (M) V.minteq	Uptake Rate (mol cell^{-1}·h^{-1})	k_{in} (L cell^{-1}·h^{-1})
Synechococcus PCC7002	A+	76	87	4.06×10^{-12}	6.11×10^{-21}	1.51×10^{-9}
	YBG11	75	88	2.74×10^{-13}	1.22×10^{-21}	4.44×10^{-9}
Synechococcus WH7803	AMP1	19.8	28.2	2.50×10^{-11}	4.37×10^{-21}	1.75×10^{-10}
	filtered sea water	22.7	30.9	5.11×10^{-11}	7.10×10^{-21}	1.39×10^{-10}

Table S1. *Cont.*

Organism	Medium	[EDTA] (μM)	[FeEDTA] (nM)	Fe' (M) V.minteq	Uptake Rate (mol cell^{-1}·h^{-1})	k_{in} (L cell^{-1}·h^{-1})
Synechococcus	AMP1	20.4	22.7	1.92×10^{-11}	2.79×10^{-21}	1.46×10^{-10}
WH8102	SOW	21.2	24	2.02×10^{-11}	3.95×10^{-21}	1.95×10^{-10}

4. Influence of Organic Buffers and pH on the Ferrozine Assay

Over the course to this study we found that the ferrozine assay is extremely sensitive to experimental conditions and results must be interpreted with care. We found that in bicarbonate buffered mediums with pH ≥ 8.2, ferrozine did not influence uptake rate. This is most likely because the formation of Fe(II)Fz_3 complex is pH sensitive (Gibbs 1976). Medium pH was therefore kept between 7.8 and 8.1. In addition, the presence of organic buffers (e.g., HEPES, TRIS) interfered with the ferrozine effect in some of the mediums. Therefore organic buffers were avoided and replaced with 2 mM $NaHCO_3$ in all mediums used in short term ^{55}Fe uptake experiments.

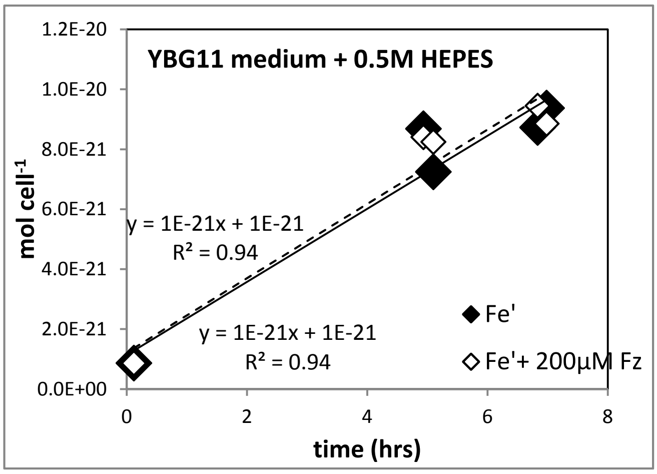

(a)

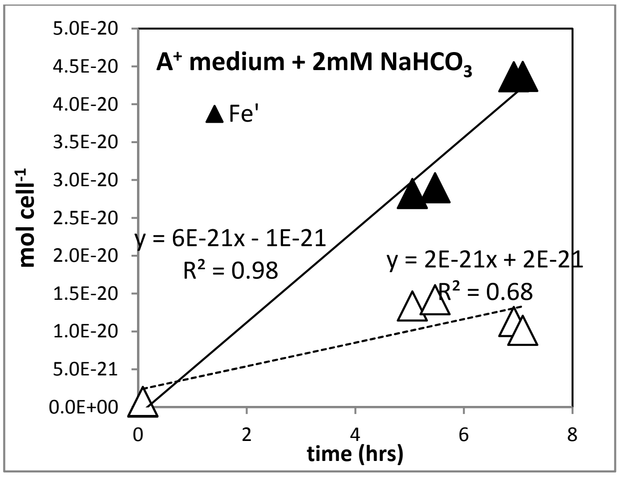

(b)

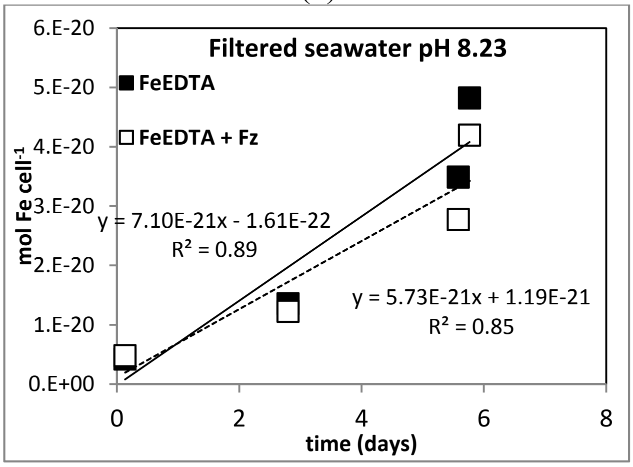

(c)

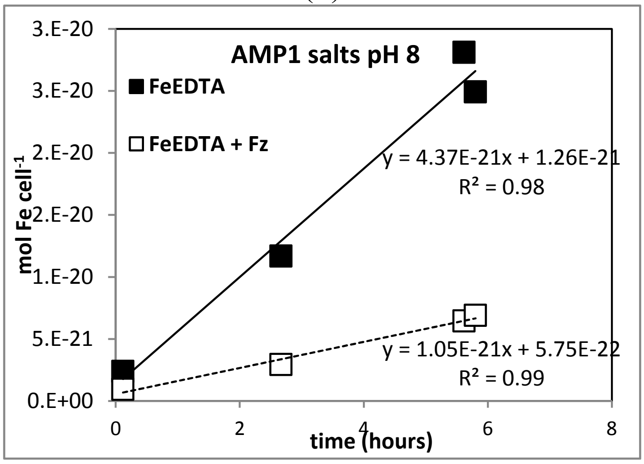

(d)

Figure S4. (**A**) ^{55}Fe' uptake by *Synechococcus* PCC7002 in uptake medium containing an organic buffer HEPES (**B**) ^{55}Fe' uptake by *Synechococcus* PCC7002 in uptake medium containing 2mM $NaHCO_3$. Both experiments were performed with the same cells and

ferrozine; (**C**) ^{55}Fe' uptake by *Synechococcus* WH7803 in uptake medium at pH 8.0 and in uptake medium at pH 8.23 (**D**). Both experiments were performed with the same cells and ferrozine.

5. Chemical characteristics of Fe chelators used in this study

Table S2. Chemical Characteristics of Fe Chelators Used in this Study.

	**Structure**	$logK_{cond}^{Fe(III)}$	**E$_{1/2}$ (mV *vs.* NHE)** [e]
EDTA	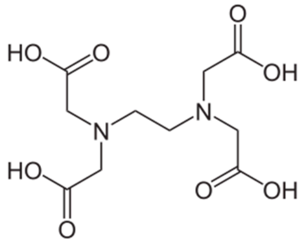	24[a]	+120
Desferrioxamine B (DFB)	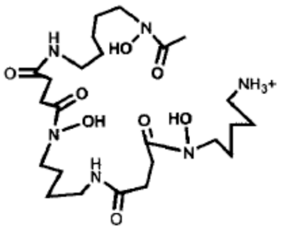	29.6 [b]	−468
Aerobactin	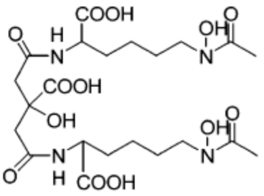	24.6 [c]	−336
Schizokinen	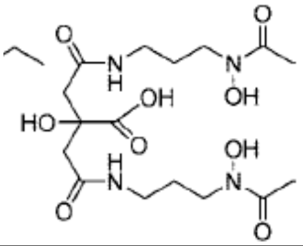	Not measured, presumably similar to that of Aerobactin	Not measured, presumably similar to that of Aerobactin
Desferrioxamine E (DFE)	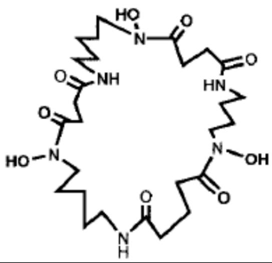	32.5 [d]	−477

a—(Rue and Bruland 1995); b—(Hudson *et al.* 1992); c—(Küpper *et al.* 2006); d—(Martell and Smith 1975); e—see (Boukhalfa and Crumbliss 2002) and references therein.

6. Fe' Uptake and Ionic Strength: Normalization on kin to Activity Co-Efficients

Short term ^{55}Fe' uptake experiments were performed in different uptake media which varied in ionic composition and ionic strength. Ionic composition is accounted for by the [Fe'] calculations performed

using Visual Minteq. However, ionic strength influences Fe activity and thus reactivity in the various mediums. We thus calculated the activity co-efficients of the dominant Fe'-species $Fe(OH)_2^+$ in accordance with Millero and Pierrot (2007). The calculated activity co-efficients are : 0.027 for SOW and AMP1; 0.165 for YBG11 and 0.050 for A+ medium. Normalization on k_{in} vales to these activity co-efficients accounted for the scatter in k_{in} values versus surface area (Figure S5).

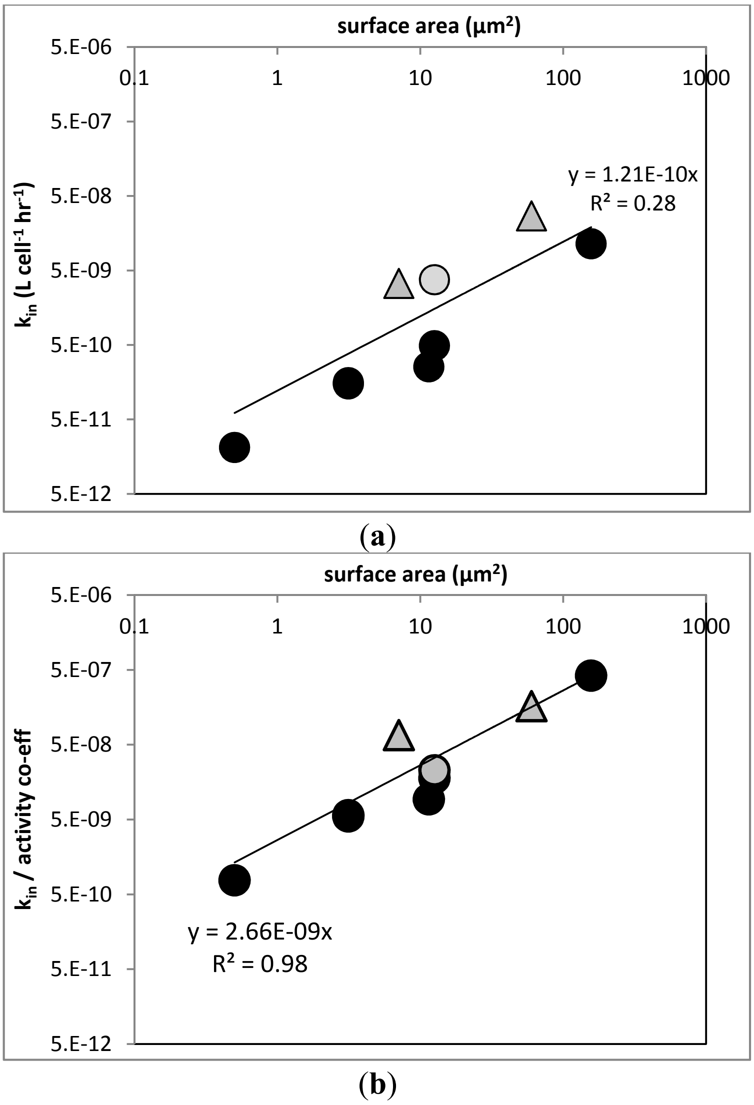

Figure S5. Fe' kin values for iron limited cyanobacteria versus cell surface are before (**A**) and after (**B**) normalization to the activity coefficient of the experimental medium. Grey symbols indicate fresh and brackish water strains while black symbols are marine strains. Triangles are siderophore producing cyanobacteria and circles are cyanobacteria which are not known to produce siderophores.

7. Presence of Siderophores in Uptake Experiments with Siderophore Producers

Anabaena UTEX2576 and *Synechococcus* PCC7002 are both known siderophore producers. All cells are thoroughly washed prior to suspension in the experimental medium and in the interpretation of short term Fe uptake experiments with these organisms we assume siderophore secretion over the experimental period is negligible. Three lines of evidence support this assumption:

(1). We established that ferrozine does not inhibit Fe-siderophore uptake in siderophore producers (see effect of ferrozine on Fe-Schizokinen uptake by Anabaena in Table 4 in the main text and on Fe-Aerobactin uptake by Anabaena and Synechococcus PCC7002 in Figure 6 in the main text). On the other hand Fe' uptake by both these organisms is significantly inhibited by the presence of ferrozine, suggesting that there is no significant concentration of siderophores in the uptake medium.

(2). The uptake rates of Fe-siderophore and Fe' are different. A comparison of Fe' and Fe-Schizokinen uptake by Anabaena is made in Figure 7 and Table 3 in the main text and Fe' is taken up 10 times faster than of Fe-schizokinen. If siderophores were present at significant concentrations in the Fe' uptake medium, we would expect to see little difference between Fe’ and Fe-schizokinen uptake rates.

(3). Surface area normalized Fe'uptake in siderophore producing organisms is very similar to that of organisms which do not produce siderophores (see Figure 3 in the main text). Since Fe-siderophore uptake rates are different to Fe’ uptake rates (as discussed above), this also supports the fact that no significant siderophore concentrations were present in the experimental medium.

8. Fe-Aerobactin Uptake

As compared to fresh and brackish water cyanobacteria in this study, the ferric aerobactin (FeAB) complex is not taken up linearly by marine cyanobacterial strains (Figure S6). Raw uptake data shows saturation and we hypothesize that FeAB is adsorbed to the cell surface rather than transported into the cell. As mentioned in the main text, FeAB has a net negative molecular charge of −3, very likely leading to repulsion of the complex from the negatively charged outer membrane of cyanobacteria. Further evidence supporting this hypothesis can be found by looking at the (a) first time point of short term uptake experiments and at (b) the signal found in the titanium wash used in these experiments.

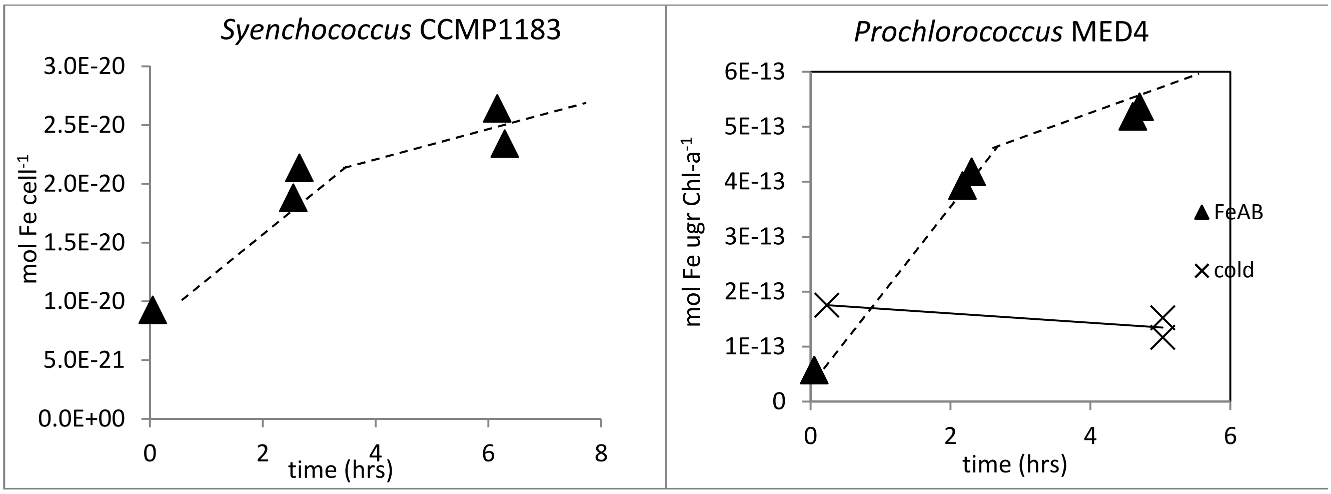

(A) **(B)**

Figure S6. *Cont.*

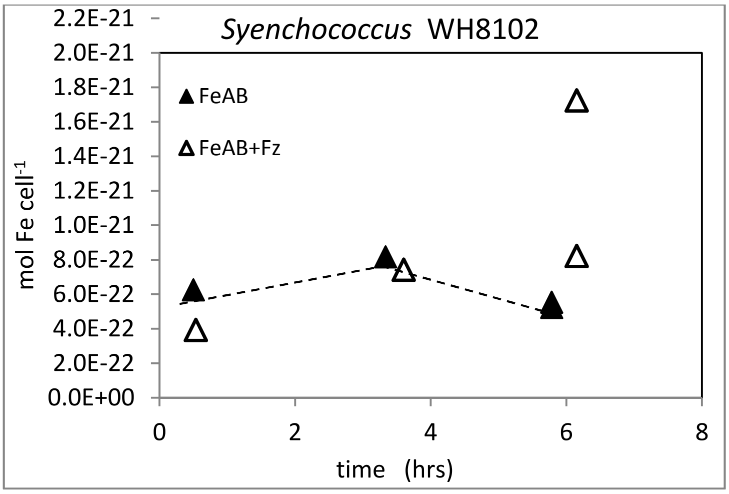

(C)

Figure S6. 55FeAB uptake by iron-limited open ocean strains (**A**) *Prochlorococcus* MED4 and (**B**) *Synechococcus* CCMP1183 and (**C**) *Synechococcus* WH8102 in the absence and presence of ferrozine.

(a) The first time point of uptake experiments represents the iron fraction which immediately binds to the cells upon their introduction into the experimental medium. As compared to Fe' and FOB, FeAB appears to be less "sticky"—showing significantly lower signals at the first time point (Figure S7).

(b) The iron fraction measured in the titanium wash represents the iron which is has "stuck" to the cell surface (the titanium wash allows the measurement of the intracellular fraction). Again, as compared to Fe'and FOB, the FeAB signals are lower, suggesting this compound is less "sticky" (Figure S8).

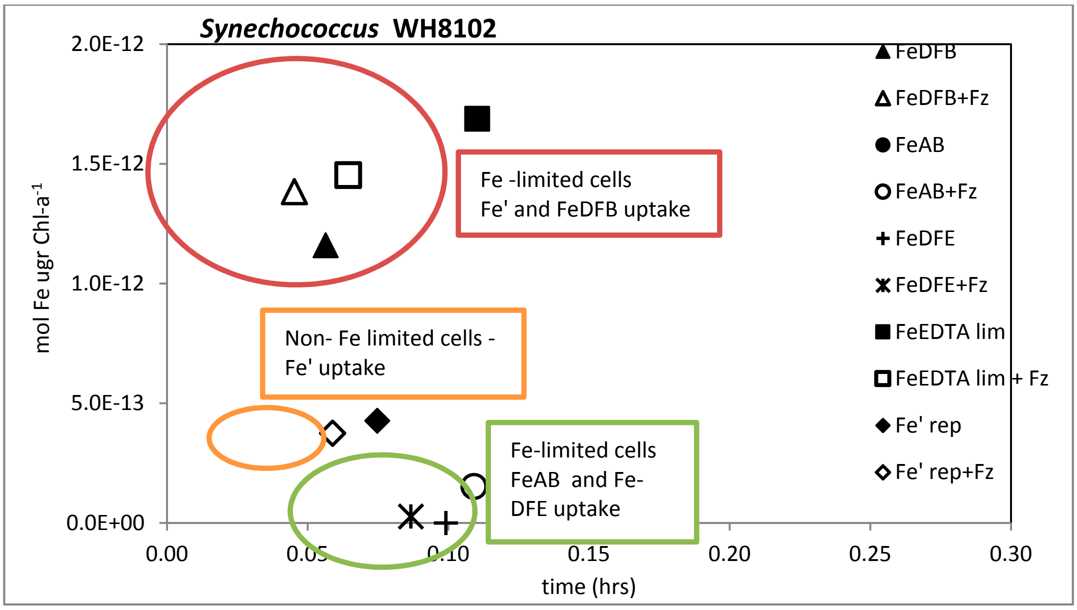

Figure S7. A comparison of the first time point in short term 55Fe uptake by *Synechococcus* WH8102. FeAB and Fe-DFE show the lowest signals while Fe' and FOB uptake by iron limited cells have the highest signal.

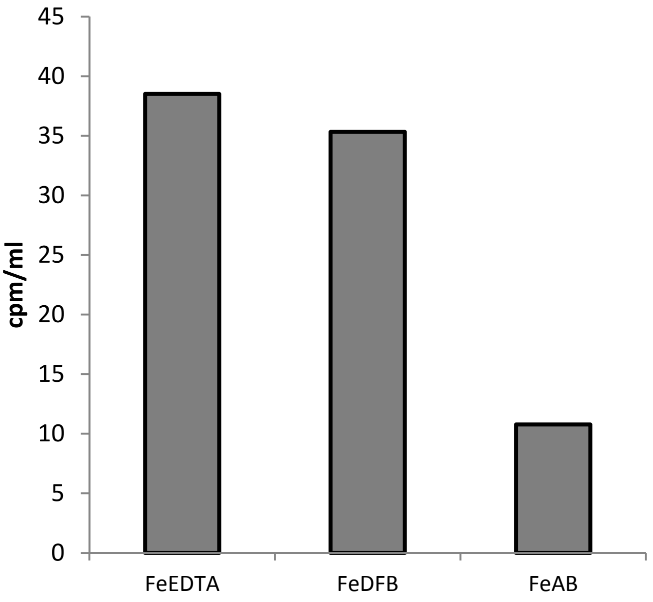

Figure S8. ^{55}Fe signal in counts per minute per mL (cpm/mL) in the titanium wash used to wash *Synechococcus* WH8102 cells in short term ^{55}Fe uptake experiments. This Fe fraction represents the iron found on the cell surface rather than inside the cell.

9. Uptake of Fe-schizokinen and Ferrioxamine E.

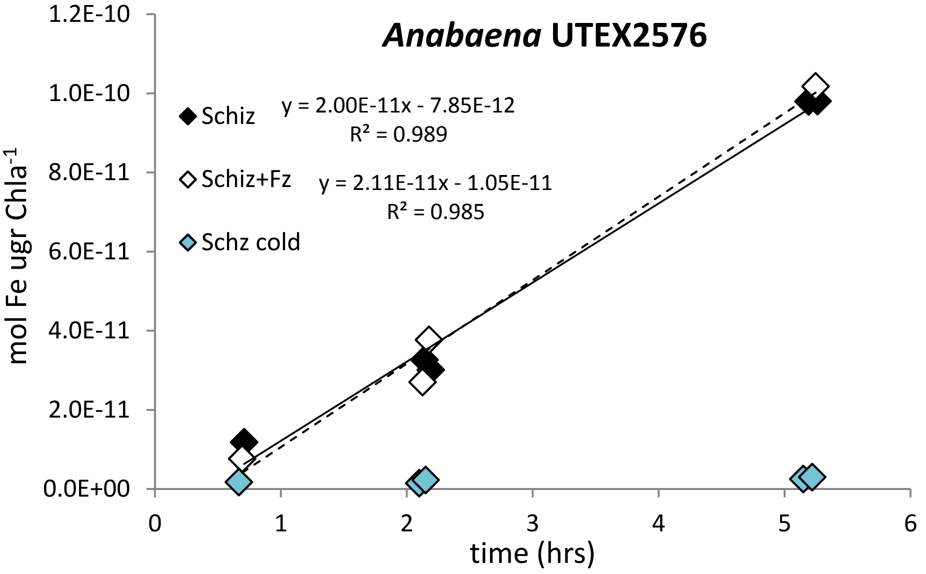

Figure S9. FeSchizokinen uptake by iron limited *Anabaena* UTEX2576 cells in the presence and absence of 200 μM ferrozine. A cold control of FeSchizokinen uptake was conducted at 4 °C.

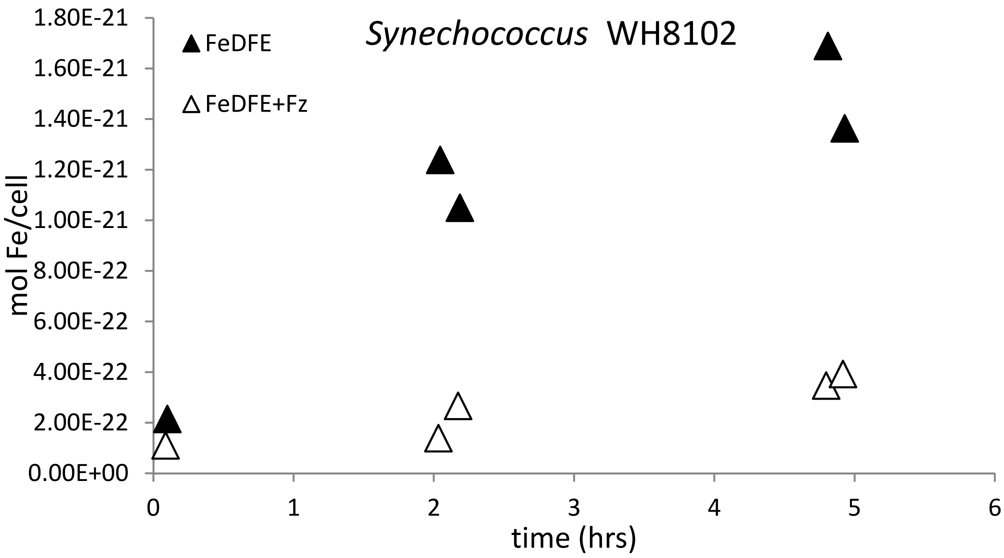

Figure S10. Ferrioxamine E (FOE) uptake by iron limited *Synechococcus* WH8102 cells in the presence and absence of 200 μM ferrozine.

10. Comparison of Fe Uptake across Substrates and Organisms

The iron uptake of different compounds by a given organism can be compared by looking at the uptake rate constants (k_{in} values), where k_{in} = uptake rate/Fe-substrate concentration. Note the values in Table S3 have not been normalized to surface area and therefore comparisons cannot be made across organisms.

Table S3. Range of k_{in} values ($L \cdot cell^{-1} \cdot hr^{-1}$) for Fe', ferrioxamine B (FOB) and ferric aerobactin (FeAB) by cyanobacteria. k_{in}= uptake rate / Fe-substrate concentration. Fe' uptake was analyzed for both iron limited and non-limited cells while FOB and FeAB uptake was measured only in iron limited cells. No uptake implies that no signal accumulated in cells over time, while experiments which were not performed are labeled with a dash (-). k_{in} values are in units of 10^{-10} (for Fe') and 10^{-14} (for FOB and FeAB). The total ^{55}Fe concentration in all uptake experiments did not exceed 90 nM.

**Organism**	**Fe' (Free Inorganic Iron)**		**FOB**	**FeAB**
	**Non-Limited**	**Fe-Limited**	**Fe-Limited**	**Fe-Limited**
	$\times 10^{-10}$	$\times 10^{-10}$	$\times 10^{-14}$	$\times 10^{-14}$
***Synechococcus* WH8102**	0.081–0.415	0.76–2.96	0.97–3.68	no uptake
***Synechococcus* WH7803**	~0.258	1.22–1.77	1.69–2.07	no uptake
***Synechococcus* CCMP1183**	~0.732	1.28–4.86	0.58–1.05	3.28–5.21
***Synechococcus* PCC7002**	1.05–5.11	15.9–51.1	1.31–2.08	~3.92
***Prochlorococcus* MED4**	0.0716–0.0977	0.124 -0.282	3.42–4.66	no uptake
***Synechocystis* PCC6803 ***	~4.55	~37.63	~6.60	~23.7
***Trichodesmium* IMS101**	10.1–13.5	~113	-	-
***Anabaena* UTEX2576**	14.4–65.0	12.7–187	37.6–62.2	14.5–18.0

* Data for Synechocystis PCC6803 was taken from Kranzler *et al.* [16]; a—data taken from Kranzler *et al.* [16];
* In FOB uptake by *Anabaena*, Fz inhibition was not significant in all experiments.

11. Media Composition

Table S4. Growth Media Composition. Below, specifies the composition of the growth media used in this study. Short term iron uptake experiments were conducted in the salt mixes of growth media (*i.e.*, without nutrients, vitamins and trace metals) and without organic buffers but rather 2 mM $NaHCO_3$. For details regarding YBG11 medium see Kranzler *et al.* (2011).

		**Aquil** *	**AMP1** §	**A+** Δ
Anhydrous salts	Na_2SO_4	2.88×10^{-2}		
	NaCl	4.20×10^{-1}	4.81×10^{-1}	3.08×10^{-1}
	KCl	9.39×10^{-3}	8.99×10^{-3}	8.05×10^{-3}
	$NaHCO_3$	2.38×10^{-3}	1.98×10^{-2}	2.38×10^{-3}
	KBr	8.40×10^{-4}		
	H_3BO_3	4.85×10^{-4}		5.54×10^{-4}
	NaF	7.15×10^{-5}		
Hydrous salts	$MgCl_2 \cdot 6H_2O$	5.46×10^{-2}	2.70×10^{-2}	
	$CaCl_2 \cdot 2H_2O$	1.05×10^{-2}	1.00×10^{-2}	1.81×10^{-3}
	$SrCl_2 \cdot 6H_2O$	6.38×10^{-5}		
	MgSO4·7H2O		2.80×10^{-2}	2.03×10^{-2}
Organic buffers	HEPES		1.00×10^{-3}	
	TRIS			8.25×10^{-3}
Nutrients & vitamins	$NaH_2PO_4 \cdot H_2O$	1.00×10^{-5}	5.00×10^{-5}	3.67×10^{-4}
	$NaNO_3$	1.00×10^{-4}		1.18×10^{-2}
	$(NH_4)_2SO_4$		4.00×10^{-7}	
	$Na_2SiO_3 \cdot 9H_2O$	1.00×10^{-4}		
	B_{12}	3.96×10^{-10}		2.95×10^{-9}
	Biotin	2.50×10^{-9}		
	Thiamine	2.96×10^{-7}		
EDTA and Trace metals	EDTA	1.00×10^{-4}	1.00×10^{-7}	8.06×10^{-5}
	Fe-$FeCl_3 \cdot 6H_2O$	variable	variable	variable
	Zn—$ZnSO_4 \cdot 7H_2O$	7.97×10^{-8}	8.00×10^{-10}	2.31×10^{-6}
	Mn—$MnCl_2 \cdot 4H_2O$	1.21×10^{-7}	9.00×10^{-9}	2.18×10^{-5}
	Co—$CoCl_2 \cdot 6H_2O$	5.03×10^{-8}	5.00×10^{-10}	5.11×10^{-8}
	Cu—$CuSO_4 \cdot 5H_2O$	1.96×10^{-8}		1.20×10^{-8}
	$Na_2MoO_4 \cdot 2H_2O$	1.00×10^{-7}	9.00×10^{-9}	1.24×10^{-7}
	Na_2SeO_3	1.00×10^{-8}	1.00×10^{-9}	
	NiCl2		1.00×10^{-9}	

* (Morel *et al.* 1979); § (Moore *et al.* 2007); Δ(Stevens *et al.* 1973).

12. Titanium Wash

The titanium-citrate-EDTA wah (Hudson and Morel 1989) was developed in order to allow the distinction between extracellular and intracellular iron. The inorganic reductant titanium (III) complexed by citrate and EDTA rapidly dissolves extracellular iron at pH 8 without causing cell breakage or toxicity in most phytoplankton species. For titanium wash components and preparation procedures see Hudson and Morel (1989).

References

1. Boukhalfa, H.; Crumbliss, A.L. Chemical aspects of siderophore mediated iron transport. *Biometals* **2002**, *15*, 325–339.
2. Gibbs, C.R. Characterization and application of ferrozine iron reagent as a ferrous iron indicator. *Anal. Chem.* **1976**, *48*, 1197–1201.
3. Guikema, J.A.; Sherman, L.A. Organization and function of chlorophyll in membranes of cyanobacteria during iron starvation. *Plant Physiol.* **1983**, *73*, 250–256.
4. Petter, G.J. Visual MINTEQ, 2010.
5. Hudson, R.J.; Covault, D.T.; Morel, F.M. Investigations of iron coordination and redox reactions in seawater using⁵⁹ Fe radiometry and ion-pair solvent extraction of amphiphilic iron complexes. *Mar. Chem.* **1992**, *38*, 209–235.
6. Hudson, R.J.; Morel, F.M. Distinguishing between extra-and intracellular iron in marine phytoplankton. *Limnol. Oceanogr.* **1989**, *34*, 1113–1120.
7. Kranzler, C.; Lis, H.; Shaked, Y.; Keren, N. The role of reduction in iron uptake processes in a unicellular, planktonic cyanobacterium. *Environ. Microbiol.* **2011**, *13*, 2990–2999.
8. Küpper, F.C.; Carrano, C.J.; Kuhn, J.-U.; Butler, A. Photoreactivity of iron (III)-aerobactin: photoproduct structure and iron (III) coordination. *Inorg. Chem.* **2006**, *45*, 6028–6033.
9. Martell, A.; Smith, R. *Critical Stability Tables*; Plenum Press: New York, NY, USA, 1975.
10. Millero, F.J.; Pierrot, D. The activity coefficients of Fe (III) hydroxide complexes in NaCl and NaClO₄ solutions. *Geochim. Cosmochim. Acta* **2007**, *71*, 4825–4833.
11. Moore, L.R. Culturing the marine cyanobacterium Prochlorococcus. *Limnol. Oceanogr. Methods* **2007**, *5*, 353–362.
12. Morel, F.M.; Rueter, J.; Anderson, D.M.; Guillard, R. AQUIL: A Chemically Defined Phytoplankton Culture Medium for Trace Metal Studies 12. *J. Phycol.* **1979**, *15*, 135–141.
13. Rue, E.L.; Bruland, K.W. Complexation of iron (III) by natural organic ligands in the Central North Pacific as determined by a new competitive ligand equilibration/adsorptive cathodic stripping voltammetric method. *Mar. Chem.* **1995**, *50*, 117–138.
14. Shaked, Y.; Kustka, A.B.; Morel, F.O.M.M. A general kinetic model for iron acquisition by eukaryotic phytoplankton. *Limnol. Oceanogr.* **2005**, *50*, 872–882.
15. Singh, A.K.; Sherman, L.A. Reflections on the function of IsiA, a cyanobacterial stress-inducible, Chl-binding protein. *Photosynth. Res.* **2007**, *93*, 17–25.
16. Stevens, S.; Patterson, C.; Myers, J. The Production of Hydrogen Peroxide by Blue - Green Algae: A Survey 1. *J. Phycol.* **1973**, *9*, 427–430.

17. Sunda, W.; Huntsman, S. Effect of pH, light, and temperature on Fe–EDTA chelation and Fe hydrolysis in seawater. Marine Chemistry **2003**, *84*, 35–47.
18. Sunda, W.G.; Price, N.M.; Morel, F.M.M. Trace metal ion buffers and their use in culture studies. *Algal Cult. Tech.* **2005**, 35–63.
19. Öquist, G. Iron Deficiency in the Blue - Green Alga Anacystis nidulans: Changes in Pigmentation and Photosynthesis. *Physiol. Plantarum* **1974**, *30*, 30–37.
